# Reply to the Comments on “GLM7—A Novel Composite Glycolipid Index Derived from Routine Health Indicators for Enhanced Diagnosis and Prediction of Multimorbidity”

**DOI:** 10.1002/advs.75147

**Published:** 2026-04-02

**Authors:** Zhihua Wang, Suowen Xu

**Affiliations:** ^1^ Department of Endocrinology and Metabolism Centre For Leading Medicine and Advanced Technologies of IHM The First Affiliated Hospital of USTC Division of Life Sciences and Medicine University of Science and Technology of China Hefei China

## Abstract

This paper is a formal response to the comments raised by Yiquan Wang et al. and Peikai Sun et al. on our published work entitled “GLM7–A Novel Composite Glycolipid Index Derived from Routine Health Indicators for Enhanced Diagnosis and Prediction of Multimorbidity”. We address all the comments in this response. In addition, we emphasize the intended application scenarios and potential limitations of GLM7 and discuss future research efforts to improve its methodological rigor and interpretability.

## 1 Introduction

We sincerely thank the authors of both comments for their interest in our work and for fostering academic discussion on the GLM7 index [1, 2, 3]. Both comments raised pertinent questions regarding the rationale for indicator selection, the annotation of disease diagnostic criteria, data dimensionality, data normalization, and missing value handling—all of which are critical for advancing the clinical and research application of this novel index. We greatly appreciate these valuable, insightful comments and the opportunity to clarify our research methodology and further refine our work.

## 2 Rationale for Indicator Selection and Diagnostic Criteria for Studied Diseases

### 2.1 Rationale for Including FBG and Other Indicators in GLM7

Wang et al. and Sun et al. both questioned the rationality of including fasting blood glucose (FBG), a well‐recognized strong correlate of diabetes, as a component of GLM7. We wish to clarify that all seven indicators constituting GLM7 were identified to be associated with the studied diseases via univariate screening, with FBG being only one of them; this multi‐indicator integration is precisely the key reason for the superior predictive performance of GLM7. Although derived from these individual clinical indicators, GLM7 maintains relative independence from its components: owing to its specific calculation methodology, it reconciles the inherent differences in units and value ranges among the seven indicators, thereby serving as a novel integrated metric designed to enhance the predictive capacity for disease diagnosis and risk stratification. Notably, traditional clinical indicators such as FBG and glycated hemoglobin (HbA1c) remain valid for disease diagnosis and reference, with their established diagnostic cut‐off values unchanged. The same rationale applies to age, a variable raised by Wang et al. For a more intuitive illustration of the contribution of each constituent indicator to GLM7 across different diseases, Figure S1 in the Supplementary Material of the original manuscript [1] elucidates the weighted importance ranking of each indicator based on SHapley Additive exPlanations (SHAP) values. It is critical to emphasize that GLM7 is an independent composite index proposed in our study; it does not contradict traditional single indicators but rather represents an integration and optimization of existing routine health indicators to improve the efficacy of disease prediction and diagnosis.

### 2.2 Diagnostic Criteria for Liver Disease

We apologize for the insufficient description of the liver disease definition in the original manuscript, which Sun et al. noted was simply stated as any physician‐diagnosed liver disorder. Due to space constraints, the operational definition of liver disease, which strictly adheres to the diagnostic criteria for liver‐related conditions specified in the National Health and Nutrition Examination Survey (NHANES) (https://wwwn.cdc.gov/Nchs/Data/Nhanes/Public/2021/DataFiles/MCQ_L.htm), was not fully elaborated. The key variables extracted from the NHANES database for liver disease screening in this study included: MCQ160L (history of any liver disease), MCQ500 (history of specific liver disease in adolescents), MCQ510A (fatty liver), MCQ510B (liver fibrosis), MCQ510C (liver cirrhosis), MCQ510D (viral hepatitis), MCQ510E (autoimmune hepatitis), and MCQ510F (other liver diseases). Our primary research objective was to capture the broad association between GLM7 and overall liver disease susceptibility; thus, no further stratified analysis was performed for specific liver disease subtypes. Accordingly, the application of the GLM7 index for liver disease is currently limited to preliminary risk prediction. Refined diagnostic stratification for specific liver disorders still requires confirmation by complementary examinations such as radiological imaging and liver histopathology. This limitation has been explicitly addressed in the Limitations section of the original manuscript: “For instance, a non‐linear association was observed between the GLM7 index and the risk of liver disease, suggesting that the impact of glycolipid metabolic disturbance on liver health may vary with different ranges of the GLM7 index. This highlights the necessity for refined interpretation of the GLM7 index rather than a “one‐size‐fits‐all” analytical approach.”

## 3 Issues Related to Data Dimensionality and Data Normalization

**FIGURE 1:**
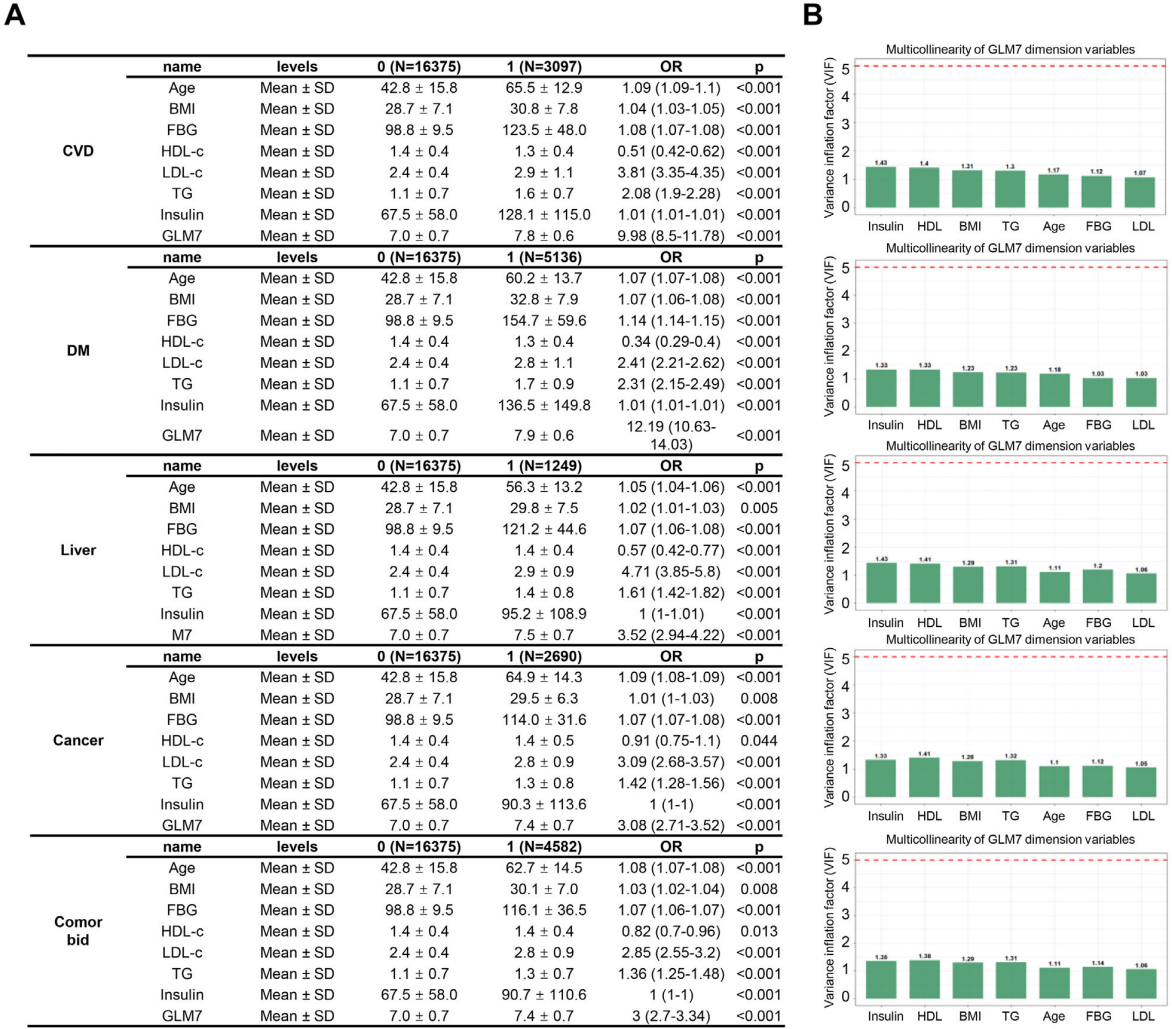
Composite dimensional and single‐dimensional analysis of GLM7 and variance inflation factor (VIF) testing. (A) Odds ratios (ORs) presented per standard deviation (SD) increase for all continuous variables across different diseases. (B) Variance inflation factor (VIF) testing validates the independence of GLM7 (GLM7 remained an independent risk factor for all studied diseases in multivariate analysis, with all VIF values < 5, indicating no significant multicollinearity).

Wang et al. and Sun et al. both noted the significant difference in odds ratios (OR) between GLM7 and age in the univariate logistic regression analysis, and this difference is indeed directly related to the dimensional definition of GLM7. GLM7 is not a single clinical indicator but a multidimensional composite index constructed from multiple routine health indicators; its dimensional design is inherently intended to quantitatively integrate multiple pathological features of disease onset and progression, rather than simply characterize a single disease‐related variable. In contrast, age is a continuous, single‐dimensional, nonspecific risk factor, whose impact on diseases such as diabetes mellitus (DM) and cardiovascular disease (CVD) is slow and indirect, and its association is diluted by other confounding factors. This explains why the OR value of age is close to 1 in univariate analysis. In contrast, the composite dimensional design of GLM7 amplifies the pathological signals directly associated with DM and CVD, resulting in a strong association (high OR value) in univariate regression. This difference is a reasonable statistical outcome driven by the specificity and integrativeness of GLM7's dimensional design, rather than a bias from statistical analysis. This is precisely the original motivation for developing the GLM7 index: to more sensitively capture the pathological association with the studied diseases. We greatly appreciate the data processing methods suggested by the authors of both comments; to better characterize this association, we have presented the ORs per standard deviation (SD) increase for all continuous variables in accordance with the suggestion of Wang et al. Additionally, we excluded the risk of multicollinearity in GLM7 via variance inflation factor (VIF) testing, effectively avoiding the pitfalls of dimensional analysis. The relevant results are presented in Figure 1.

## 4 Access to CHARLS Data and Imputation of Missing Insulin Values

**FIGURE 2:**
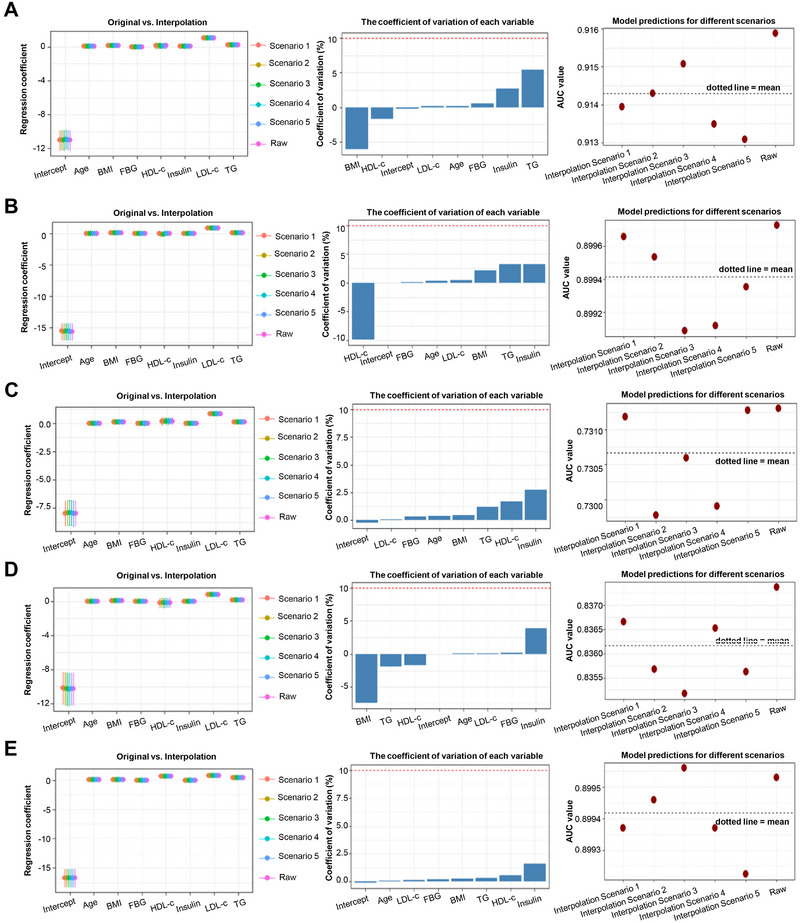
Sensitivity analysis using the MICE method for multiple imputation. (A–E) Show the distribution of the coefficient of variation (CV, %) of variables and the range of area under the curve (AUC) of related predictive indicators under different imputation models for cardiovascular diseases, diabetes, liver diseases, cancer, and comorbidities. If the CV% of all variables is less than 10% and the fluctuation of AUC is less than 0.05, then the original imputation results are considered reliable, and the model conclusion is also credible.

As noted by Sun et al. in their comment, data from the China Health and Retirement Longitudinal Study (CHARLS) are available upon registered application via the official website (https://charls.charlsdata.com/pages/data/111/zh‐cn.html), and data processing must comply with the official usage regulations and manual [4, 5]. For the systematic missing of insulin values in the CHARLS dataset, we adopted multiple imputation, a method routinely accepted in the statistical community and by top medical journals (e.g., The New England Journal of Medicine, The Lancet, BMJ) [6, 7, 8, 9]. We apologize for the omission of this methodological detail in the original manuscript due to space constraints. Briefly, our imputation approach followed the methods described by Held U et al. and Vergouwe Y et al. [10, 11]: first, the NHANES and CHARLS datasets were vertically concatenated to form a single integrated dataset; then, the Multivariate Imputation by Chained Equations (MICE) package [12] was used to predict the missing insulin values in CHARLS, with the seven routine health indicators available in both NHANES and CHARLS as predictor variables. The MICE algorithm automatically constructs a regression model with the formula: Insulin ∼ TG + TC + LDL + HDL + age + FBG + database. Critically, this model was trained on samples with known insulin values (NHANES) and then applied to predict insulin values for samples with missing data (CHARLS). The MICE algorithm was run multiple times (m = 5, maxit = 50, method = “pmm” in our study), generating m complete imputed datasets. In each dataset, the missing insulin values in CHARLS were filled, with slight variations in the imputed values across datasets to reflect the uncertainty of prediction. Subsequently, predictive models were constructed on each of the m imputed datasets separately, and the results of these m models were pooled in accordance with Rubin's Rules [13] to generate a final comprehensive result that accounts for imputation uncertainty. To evaluate the impact of the imputation method on the external validation results, we additionally performed a sensitivity analysis to clarify the contribution of insulin imputation to the predictive performance of GLM7 (Figure 2). It is important to emphasize that the external validation of GLM7 in this study is an approximate validation based on imputed data, and the interpretation of results should take into account the potential bias introduced by data imputation.

## 5 Regarding the Marginal Relationship Between *p*‐Values and Confidence Intervals

We sincerely appreciate the valuable comment by Sun et al., pointing out that some indicators in our study (e.g., HDL‐c, age) show marginal significance with *p*‐values close to 0.05. Indeed, after repeated checking of the analytical results, we confirmed the marginal significance of these parameters. In our interpretation, HDL‐c and age are non‐specific risk factors for diabetes and cardiovascular diseases, and their marginal significance represents an objective statistical observation in our study population. In particular, as a continuous non‐specific variable, age is affected by multiple confounding factors such as lifestyle, comorbidities, and metabolic status in the study population, which may weaken the strength of its association with the study outcome and consequently lead to a *p*‐value near 0.05. This result is consistent with the clinical and epidemiological characteristics of age as a disease risk factor, and its marginal significance reflects the real nature of the dataset [14, 15]. We are extremely grateful to Sun et al. for their insightful suggestions and for guiding and planning our future work direction.

In summary, we greatly appreciate the recognition and insightful comments from Wang et al. and Sun et al. on our work. Their comments have significantly improved the methodological standardization and overall quality of our study, and provided valuable guidance for the subsequent optimization, improvement, and future application of the GLM7 index. We sincerely thank them for their constructive comments and suggestions.

## Conflicts of Interest

The authors declare no conflicts of interest.

## Supporting information




**Supporting File**: advs75147‐sup‐0001‐SuppMat.docx.

## Data Availability

Data sharing not applicable to this article as no datasets were generated or analysed during the current study.
