# Supplementary material for: Reply to the Comments on “GLM7—A Novel Composite Glycolipid Index Derived from Routine Health Indicators for Enhanced Diagnosis and Prediction of Multimorbidity”
Source: Adv Sci (Weinh). 2026 Apr 2;13(27):e75147. doi: 10.1002/advs.75147 (PMC13170239; doi:10.1002/advs.75147)
Supplement: Supplementary file 1 — Supporting File: advs75147‐sup‐0001‐SuppMat.docx. [file ADVS-13-e75147-s001.docx]

**
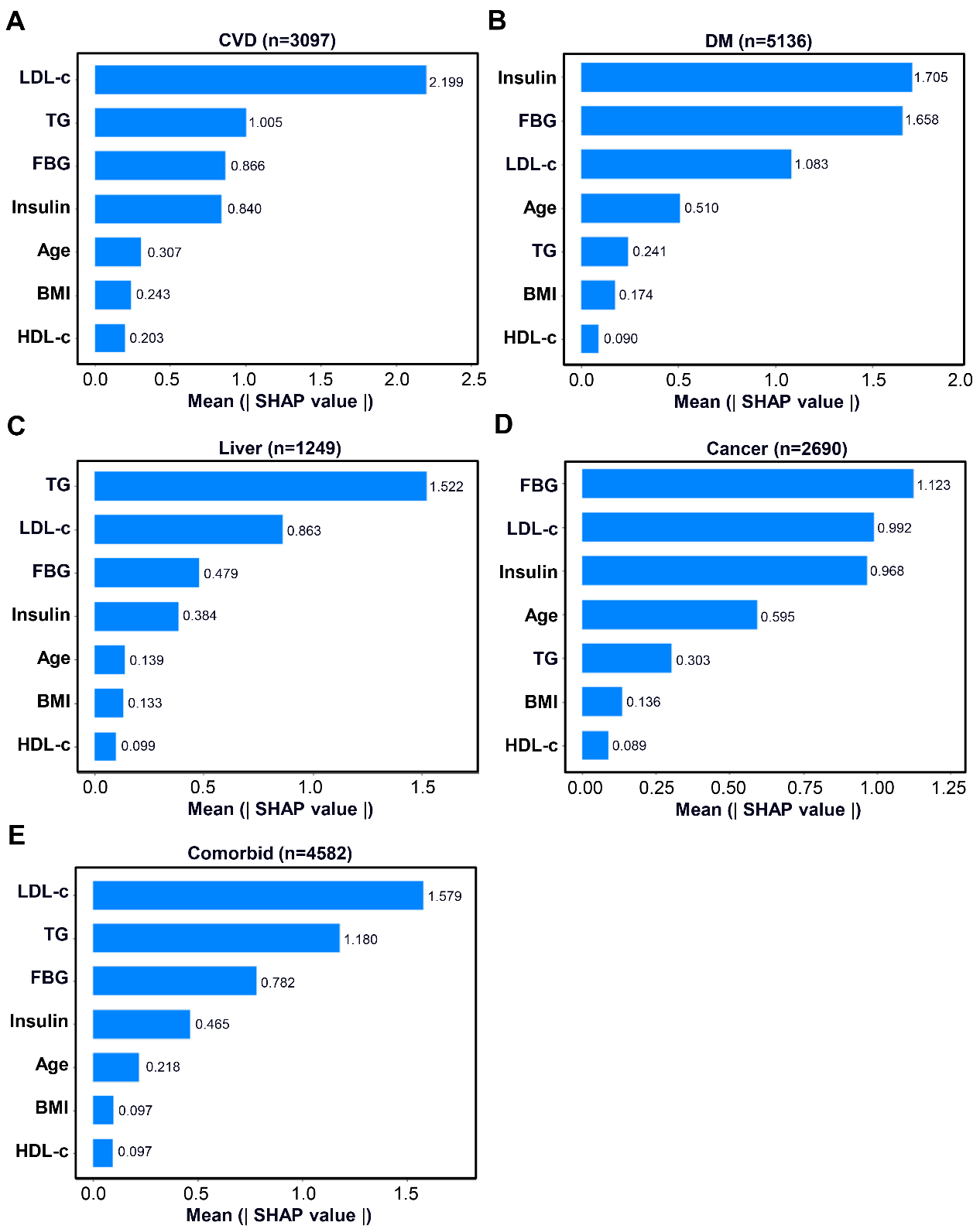
**

**Figure S1. SHAP value-based interpretation of factor weights in the GLM7 prediction model for different disease outcomes. (A)** Bar plot illustrating the SHAP values of seven factors in the GLM7 model for predicting cardiovascular disease. **(B)** Bar plot illustrating the SHAP values of seven factors in the GLM7 model for predicting diabetes mellitus. **(C)** Bar plot illustrating the SHAP values of seven factors in the GLM7 model for predicting liver diseases. **(D)** Bar plot illustrating the SHAP values of seven factors in the GLM7 model for predicting cancer. **(E)** Bar plot illustrating the SHAP values of seven factors in the GLM7 model for predicting comorbidities.
